# Bioconversion of paper sludge to biofuel by simultaneous saccharification and fermentation using a cellulase of paper sludge origin and thermotolerant *Saccharomyces cerevisiae *TJ14

**DOI:** 10.1186/1754-6834-4-35

**Published:** 2011-09-29

**Authors:** Joni Prasetyo, Kazuya Naruse, Tatsuya Kato, Chuenchit Boonchird, Satoshi Harashima, Enoch Y Park

**Affiliations:** 1 Laboratory of Biotechnology, Integrated Bioscience Section, Graduate School of Science and Technology, Shizuoka University, 836 Ohya, Suruga-ku, Shizuoka 422-8017, Japan; 2 Laboratory of Biotechnology, Faculty of Agriculture, Department of Applied Biological Chemistry, Shizuoka University, 836 Ohya, Suruga-ku, Shizuoka 422-8017, Japan; 3 Department of Biotechnology, Faculty of Science, Mahidol University, Rama VI Road, Bangkok 10400, Thailand; 4 Department of Biotechnology, Graduate School of Engineering, Osaka University, Osaka, Japan

## Abstract

**Background:**

Ethanol production from paper sludge (PS) by simultaneous saccharification and fermentation (SSF) is considered to be the most appropriate way to process PS, as it contains negligible lignin. In this study, SSF was conducted using a cellulase produced from PS by the hypercellulase producer, *Acremonium cellulolyticus *C-1 for PS saccharification, and a thermotolerant ethanol producer *Saccharomyces cerevisiae *TJ14 for ethanol production. Using cellulase of PS origin minimizes biofuel production costs, because the culture broth containing cellulase can be used directly.

**Results:**

When 50 g PS organic material (PSOM)/l was used in SSF, the ethanol yield based on PSOM was 23% (g ethanol/g PSOM) and was two times higher than that obtained by a separate hydrolysis and fermentation process. Cellulase activity throughout SSF remained at around 60% of the initial activity. When 50 to 150 g PSOM/l was used in SSF, the ethanol yield was 21% to 23% (g ethanol/g PSOM) at the 500 ml Erlenmeyer flask scale. Ethanol production and theoretical ethanol yield based on initial hexose was 40 g/l and 66.3% (g ethanol/g hexose) at 80 h, respectively, when 161 g/l of PSOM, 15 filter paper units (FPU)/g PSOM, and 20% inoculum were used for SSF, which was confirmed in the 2 l scale experiment. This indicates that PS is a good raw material for bioethanol production.

**Conclusions:**

Ethanol concentration increased with increasing PSOM concentration. The ethanol yield was stable at PSOM concentrations of up to 150 g/l, but decreased at concentrations higher than 150 g/l because of mass transfer limitations. Based on a 2 l scale experiment, when 1,000 kg PS was used, 3,182 kFPU cellulase was produced from 134.7 kg PS. Produced cellulase was used for SSF with 865.3 kg PS and ethanol production was estimated to be 51.1 kg. Increasing the yeast inoculum or cellulase concentration did not significantly improve the ethanol yield or concentration.

## Background

Recently, much research has been conducted on reducing the input energy and cost of ethanol production. Around 5 million tons of paper sludge (PS) is discharged annually by the paper manufacturing industry in Japan. Disposing of PS in landfill or by incineration creates environmental problems, and legislative trends in many countries are restricting the amount and types of materials that are permitted to be disposed of by landfill [1]. The production of bioethanol from PS can reduce dependence on fossil fuels while simultaneously solving the environmental problems associated with PS disposal. The use of bioethanol produced from PS offers an alternative source of energy, which could help overcome the current fossil fuel crisis and slow global warming. Using industrial waste materials as raw materials for bioethanol production is increasingly being researched [2,3], due to the lower costs of raw materials and to avoid competition with human needs occurring when food crops are used, as is the case for first generation production processes.

Recent research into ethanol production from PS has been reported, using pretreatments such as mechanical grinding or phosphoric acid swelling to improve saccharification yield and efficiency [4]. To remove hemicelluloses in the lignocellulosic material contained in recycled PS and cotton gin waste, mixing with steam treatment has been described as an effective pretreatment. However, this pretreatment method generated compounds that are toxic to the microorganism responsible for fermentation. Some inhibitors, such as furfural and hydroxymethylfurfural that are derivatives of lignin, significantly influence the performance of cellulase and ethanol fermentation by yeast [5,6]. By using recycled PS that contains calcium carbonate (overliming), the toxic compounds can be eliminated [7]. An advantage of PS as a carbon source over other lignocellulosic materials in bioethanol production is that pretreatment is not required, since most of the lignin has already been removed during the pulping that forms part of the paper manufacturing process.

The conventional yeast used in anaerobic alcohol fermentation releases 8.1 kJ/C mol glucose and cannot degrade xylose [8], which constitutes more than 10% of the reducing sugars (RS) contained in PS. When carrying out the process on an industrial scale, the bioreactor culture temperature must be controlled using cooling water. Using thermotolerant yeast reduces the costs involved in cooling the fermentation, as well as costs associated with the distillation of ethanol.

Ethanol concentration is an important factor of biofuel production, and should be at least 40 g/l in order to decrease the energy required during the ethanol separation and purification processes [9]. In order to achieve ethanol concentrations of 40 g/l, research has been conducted into enabling ethanol production in semicontinuous fed-batch reactors. Starting ethanol concentrations of about 20 g/l have been reported, with the concentration reaching 40 g/l after 36 h [10]. Solid-state fed-batch fermentation processes conducted in a rotary drum have been shown to be an alternative method, and the gas phase containing ethanol was collected as its condensate at -10°C [11].

Most ethanol production from cellulosic biomass has been conducted using commercial cellulases. However, the potential to use PS as a carbon source using a cellulase produced by *Acremonium cellulolyticus *has already been shown [12]. This fresh cellulase, which was produced using PS as carbon source can be used directly to hydrolyse PS organic material (PSOM) that contains cellulose and hemicellulose. Only simple separation processes to remove insoluble materials such as clay and other biomass are required. In the present study, we established efficient bioethanol production using cellulase produced from PS and thermotolerant *Saccharomyces cerevisiae *TJ14 in a simple process without any pretreatment of PS. To allow for comparison, Solka Floc (SF), which is composed entirely of cellulose, was used in separate hydrolysis and fermentation (SHF) and simultaneous saccharification and fermentation (SSF) processes for ethanol production. The performance of the process was evaluated and optimized to achieve a high ethanol concentration from PS for use as a biofuel.

## Methods

### Raw materials

PS was provided by Tomoegawa Co. Ltd. (Shizuoka, Japan). The PS was collected from a primary clarifier sludge dewatering process used for the production of virgin wood fiber, which was a mixture of pine, cypress and eucalyptus. This PS contained 65% water, 10.5% clay, 24.5% organic material and 1.2 mg RS/g wet PS on a weight basis [1]. The clay composition is shown in detail in Table 1[13]. Dry PS contained 30% clay and 66% organic material consisting of cellulose and hemicellulose. Glucan and mannan levels in the hydrolysate of this PS organic material (PSOM) were 64.5% and 2.5%, respectively (Table 2). SF (CAS #9004-34-6; International Fiber Co., New York, NY, USA) was used as positive control for cellulose. SF is a fine white powder comprised of approximately 70% to 80% crystalline cellulose and 20% to 30% amorphous cellulose. Acremozyme cellulase was purchased from Meiji Seika Kaisha, Ltd. (Tokyo, Japan). Glucose, xylose, mannose and other chemicals were purchased from Wako Pure Chem. Co. Ltd. (Tokyo, Japan) and stored at room temperature.

**Table 1:** Chemical composition of representative paper sludge (PS) ash [13]

Ash	Composition (% w/w)
SiO_2_	35.7
TiO_2_	1.2
Al_2_O_3_	26
FeO^a^	0.4
MnO	0
MgO	8
CaO	25.7
Na_2_O	0.1
K_2_O	0.1

^a^Total iron as FeO.

**Table 2:** Composition of dry paper sludge (PS)

Component	Amount (g/g dry PS)
Total sugar	0.66
Glucan	0.44
Mannan	0.02
Xylan	0.07
Other sugars	0.13
Clay	0.30
Others	0.04

### Microorganisms

*A. Cellulolyticus *C-1 (Ferm P-18508), which is a hypercellulase producer and a mutant of wild-type *A. cellulolyticus *Y-94, was provided by Tsukishima Kikai Co. Ltd. (Tokyo, Japan) [14]. *A. cellulolyticus *produces a complex mixture of cellulases, mainly comprised of 4 β-glucosidases (EC 3.2.1.21) and 12 distinct endocellulase/carboxymethyl cellulase (CMCases, EC 3.2.1.4) [15,16]. Other polysaccharide hydrolyzing enzymes, such as xylanases, amylases and β-1,3-glucanases, were also present [14]. The most important enzyme in this mixture with regard to the current process is an endocellulose type III-A that can produce glucose from cellulose with no involvement of β-glucosidase [16].

A thermotolerant strain of *S. cerevisiae*, TJ14 [17], was used in this study. *S. cerevisiae *TJ14 is a hybrid strain between the heat-tolerant strain HB8(RI)-3A (*MAT*a*his3Δ1 leu2Δ0 ura3Δ0*) and an ethanol producer yeast TISTR5056, generated by spore-to-cell mating. HB8 (RI)-3A is a derivative strain from a natural thermotolerant yeast isolate (C3723) found in Thailand and a thermosensitive laboratory yeast strain BY4742 (*MAT*a*his3Δ1 leu2Δ0 lys2Δ0 ura3Δ0*) [18]. *S. cerevisiae *TJ14 can be precultivated aerobically by shaking at 200 rpm [12].

### Fermentation media and cultivations

The preculture medium for *A. cellulolyticus *consisted (per liter) of 40 g SF, 24 g of KH_2_PO_4_, 1 ml of Tween 80 (MP Biomedicals, Solon, OH, USA), 5 g of (NH_4_)_2_SO_4_, 4.7 g of K_2_C_4_H_4_O_6_·4H_2_O, 1.2 g of MgSO_4_·7H_2_O, 10 mg of ZnSO_4_·7H_2_O, 9.28 mg of MnSO_4_·7H_2_O, 8.74 mg of CuSO_4_·7H_2_O and 2 g of urea (pH 4.0). The medium was sterilized at 121°C for 20 min, with ZnSO_4_·7H_2_O, MnSO_4_·7H_2_O and CuSO_4_·7H_2_O sterilized separately. Urea was sterilized by filtering through a 0.45 μm filter membrane (Toyo Roshi Kaisha Co. Ltd., Tokyo, Japan). The cellulase production medium was comprised of 70 g PSOM/l as carbon source without the addition of any further minerals other than those contained in PS. KH_2_PO_4 _and urea were added at final concentrations of 10 g/l and 4 g/l, respectively. Cultures were conducted in a 3 l jar fermenter equipped with a Labo-controller (MDL-80, Marubishi, Tokyo Japan) with a 1.2 l working volume. The culture broth was centrifuged at 9,447 *g *and the supernatant was stored in a 4°C refrigerator. The activity of the cellulase was analyzed before use in the enzymatic hydrolysis of PSOM.

The inoculums of *S. cerevisiae *TJ14 was carried out in 50 g/l yeast/peptone/dextrose (YPD) medium containing less than 0.04% of adenine (Sigma-Aldrich Co. Ltd., St Louis, MO, USA). The YPD medium was composed of 20 g/l of bacteriological peptone, 10 g/l of yeast extract and 20 g/l of glucose. This seed culture was incubated for 24-30 h and by this time the cell density was about 2.2 to 2.8 g dry cell weight (DCW)/l. The fermentation was carried out by adding 10% (v/v) inoculum. The ethanol production medium was comprised (per liter) of 4 g KH_2_PO_4_, 2.5 g (NH_4_)_2_SO_4_, 0.6 g MgSO_4_·7H_2_O, 2.35 g K_2_C_4_H_4_O_6_·4H_2_O, 1.0 g CaCl_2_·2H_2_O, 5 g yeast extract and 10 g of polypeptone. Glucose was used as a carbon source during fermentation. In the case of ethanol production from PS, the medium was comprised (per liter) of PS, 5 g yeast extract, 10 g of polypeptone and 4 g KH_2_PO_4 _in 0.2 M maleic buffer. The quantity of PS used was varied for each experiment.

### Optimization of saccharification

PSOM was hydrolyzed in 500 ml Erlenmeyer flasks in a reciprocal shaker at an agitation rate of 110 rpm for 120 h at 42°C in 0.8 M maleate buffer with initial pH 5.2 [1]. The PSOM concentrations were varied 10, 30, 50, 70, 90, 110 g/l in maleate buffer. For the saccharification reaction, the Acremozyme cellulase (Meiji Seika Kaisha) used had an activity of 5, 10, 20, 40, 60, 80, 100 filter paper units (FPU)/g PSOM. Samples were taken every 12 h and centrifuged at 9,447 *g *for 5 min. The reaction was stopped by boiling the samples for 5 min and then measuring the RS content of the supernatant. Data were analyzed by Design Expert (v. 7.1.6, Stat-Ease, Minneapolis, MN, USA).

The percent saccharification yield (*Y*_s_) of substrates in terms of the RS concentration was calculated by the formula:

$$Y_{s}=\frac{\text{Reducing sugar concentration (g/l) }\times \text{ Hydrolysate volume (l)}}{\text{PS weight (g)}\times \text{PSOM content in the PS (\% w/w)}}\times 100$$

using a PSOM content of 24.5%.

### Separate hydrolysis fermentation and simultaneous saccharification fermentation

SHF involves enzymatic hydrolysis and fermentation, and these were carried out in 500 ml Erlenmeyer flasks with a working volume of 100 ml. The PSOM was hydrolysed by cellulase produced from PSOM as carbon source until a maximum RS concentration was achieved. A total of 5 g/l of yeast extract, 10 g/l polypeptone and 4 g/l KH_2_PO_4 _were added to the hydrolysate and this mixture was used as the fermentation medium. The medium was also sterilized to deactivate the cellulase prior to fermentation. After the sterilized medium had been cooled to 42°C, it was inoculated with 10% (v/v) of the yeast preculture and incubated with agitation at 50-80 strokes per min (spm) in a reciprocal shaker (Bioshaker TA-25R, Takasaki Scientific Instruments, Saitama, Japan) and 42°C.

**Figure 1:**
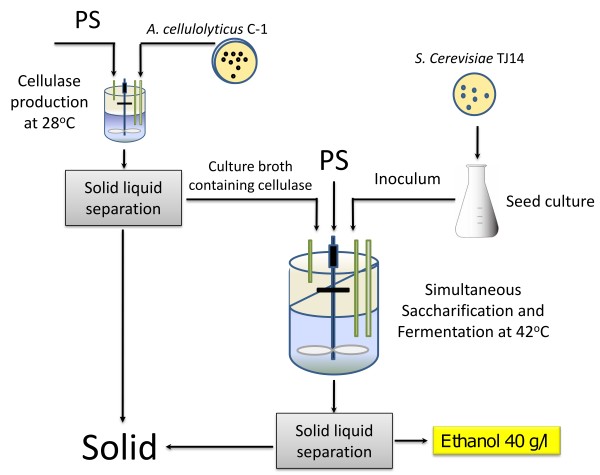
**Schematic diagram of simultaneous saccharification and fermentation (SSF) using cellulose of paper sludge (PS) origin and thermotolerant *Saccharomyces cerevisiae *TJ14**. PS was used as a carbon source for cellulase production by *Acremonium cellulolyticus *C-1 at 28°C. The culture broth containing cellulase was separated from the *A. cellulolyticus *culture and used for saccharification of PS in SSF at 42°C. Ethanol fermentation was carried out by inoculation with yeast simultaneously with saccharification of PS during SSF. After SSF, the ethanol solution was separated from the SSF culture broth.

A schematic diagram of SSF using PS as the carbon source for cellulase production and as a substrate for saccharification by the cellulase produced is shown in Figure 1. PSOM was used as a carbon source for cellulase production by *A. cellulolyticus *C-1 at 28°C. The culture broth containing the cellulase was separated from the *A. cellulolyticus *culture, and used for saccharification of PS in SSF at 42°C. In SSF, ethanol fermentation was carried out simultaneously with saccharification of PS by inoculation with yeast. Medium compositions (per liter) for SSF consisted of PSOM, 5 g yeast extract, 10 g polypeptone and 4 g KH_2_PO_4_.

For improving ethanol concentration, PSOM concentration and cellulase activity were optimized using the following conditions: initial PSOM concentrations were 50, 80 and 110 g/l and cellulase activities were 15, 25 and 35 FPU/g PSOM. After medium sterilization, the cellulase and 10% inoculum were added to 500 ml Erlenmeyer flasks with final working volumes of 100 ml. When 170 g/l of PSOM and 35 FPU/g PSOM were used, the culture could not be readily mixed. To avoid the mixing problem, the PSOM was added at 0 and 8 h of culture time as follows: 8.5 g of PSOM (PS 34.7 g containing 22.5 ml of water), 14 ml of cellulase solution, 10 ml of inoculum, and 21 ml of buffer (total working volume of 67.5 ml), and at the culture time of 8 h, another 8.5 g PSOM (PS 34.7 g containing 22.5 ml of water) and 10 ml of cellulase solution were added. The final working volume was 100 ml. The PSOM concentration was 126 g/l at 0-8 h of culture time, and 170 g/l after 8 h of culture time.

To improve ethanol production in SSF, the amount of PSOM was increased with 15 FPU/g PSOM of cellulase. Due to mixing problems, the initial concentration was 80 g PSOM/l, and 11 g PSOM (44.9 g PS) and PS cellulase 15 FPU/g PSOM were added on one, two or three occasions at culture times of 8 h, 16 h, and 20 h, respectively. The final PSOM concentration was therefore 127, 151 and 165 g PSOM/l in each case. The SSF was conducted at 42°C until a maximum ethanol concentration was reached with agitation at 50-80 spm in a reciprocal shaker (Bioshaker TA-25R, Takasaki Scientific Instruments).

To improve ethanol yield, the inoculum was increased from 10% to 20% when the following conditions were used: 100 g/l of initial PSOM with 15 FPU/g PSOM, then 11 g PSOM added at culture times of 8 and 16 h. All PS used in the above experiments was sterilized to avoid contamination in the fermentation, and fermentation was stopped when the ethanol concentration reached a maximum. After SSF, the ethanol solution was separated from insoluble material of SSF culture broth. The supernatant was refrigerated at 4°C for measurement of RS, glucose, ethanol concentrations and remaining cellulase activity.

Scale up of SSF was carried out in 2 l Erlenmeyer flask with a working volume of 1.2 l. The initial composition of SSF was 100 PSOM g/l and 15 FPU/g PSOM, and the SSF was started with 20% inoculum in 600 ml. An additional 66 g of PSOM and 15 FPU/g PSOM of cellulase were added twice at culture times of 8 h and 16 h, and then final working volume adjusted to 1.2 l.

Since the consumed concentrations of hexose and PSOM during the reaction were unknown, theoretical ethanol yield based on initial hexose (*Y_e/hex_*) was defined as follows:

$$Y_{e∕hex}=\frac{\Delta C_{e}}{C_{e∕hex}}\times 100$$

where Δ*C_e _*indicates produced ethanol concentration during the process. *C_e/hex _*indicates theoretical ethanol concentration converted from hexose containing in PSOM as follows:

$$C_{e∕hex}=0.66\times C_{psom}\times 1.11\times 0.51$$

where *C_psom _*denote initial PSOM concentrations (g/l). Constants 1.11 and 0.51 denote coefficients from hydrolysis of glucan and from hexose to ethanol, respectively.

When consumed glucose concentration (Δ *C_glc_*) was measured, theoretical ethanol yield (*Y_e/glc_*) was defined as follows:

$$Y_{e∕glc}=\frac{\Delta C_{e}}{\Delta C_{glc}\times 0.51}\times 100$$

For the practical ethanol production from PS, the ethanol yield based on the initial PSOM (*Y_e/psom _*) is as follows:

$$Y_{e∕psom}=\frac{\Delta C_{e}}{C_{psom}}\times 100$$

### Analysis methods

In the case of soluble substrate, the DCW of the microorganism was determined by centrifuging the cell broth at 5,000 *g *for 15 min. The harvested cells were resuspended in distilled water and centrifuged again to remove medium components [19]. The precipitate was dried at 105°C. In the case of PS that contained insoluble material, viable cell numbers were determined by counting colony-forming units (CFU) on an agar plate containing 1.5% agar. The CFU was converted to DCW (1.6 × 10^7 ^viable cell/g DCW) using a calibration curve. Due to the difficulty in separating the mycelia of *A. cellulolyticus *C-1 from the medium, intercellular nucleic acid concentration (*INA*) was measured and converted to dry cell weight (DCW) as follows [17]"

$$\begin{array}{c} INA\left(\text{g}∕\text{l}\right)=1.72\times \text{absorbance at}\;260\;\text{nm}\\ \text{DCW (g/l)}=16.565\times INA \end{array}$$

Cellulase activity was measured using the standard International Union of Pure and Applied Chemistry (IUPAC) procedure with Whatman no. 1 filter paper, and the activity was expressed in FPU. The FPU unit is based on the International Unit (IU) in which the absolute amount of glucose at a critical dilution is 2 mg for 0.5 ml critical enzyme concentration in 60 min [20].

The monosaccharide content was analyzed by high-performance liquid chromatography (HPLC; PU-980; JASCO Co. Ltd., Tokyo, Japan). Detection was carried out using a refractive index detector (RI-930, JASCO) and an amine-modified silica column (Shodex Asahipack NH2P-50 4E, 4.6 diameter, 250 mm, Shimadzu GLC Ltd., Tokyo, Japan) in combination with a precolumn. The mobile phase was 75% acetonitrile, and the flow rate was 1 ml/min. The total sugar content of PS was determined according to the standard National Renewable Energy Laboratory (NREL) method [21]. PS was dried at 80°C and treated with 72% H_2_SO_4 _for 1 h at 30°C, then diluted with 4% H_2_SO_4 _and autoclaved for 1 h at 121°C. Glucose and mannose concentrations were analyzed with Megazyme kits (Biocon (Japan) Ltd., Nagoya, Japan) while the RS content of the medium was determined by the dinitrosalicylic acid method.

Ethanol concentration was measured using gas chromatography (GC) (Shimadzu-2014, Shimadzu Co. Ltd., Tokyo, Japan) using a packed column (Gaskuropack 54 60/80, GC-2014 Glass ID. 3.2 diameter × 2.1 m, GL Science Co. Ltd., Tokyo, Japan), with the following operational conditions: temperature of column and detector were 110°C and 250°C, respectively; nitrogen gas flow rate was 60 ml/min; injected sample volume was 2 μl.

## Results

### Ethanol production from monosaccharide

**Figure 2:**
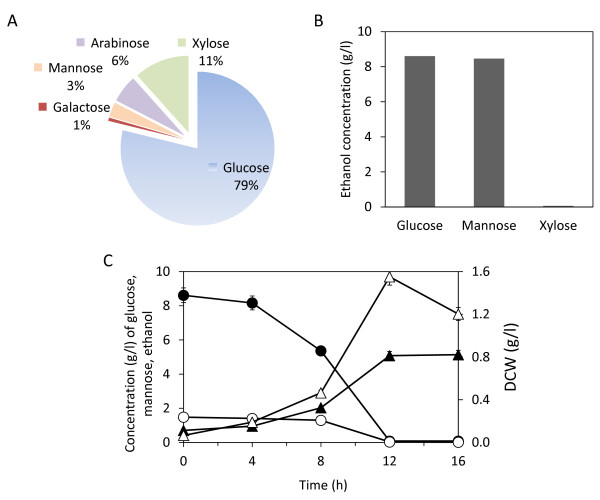
**Enzymatic hydrolysis of paper sludge (PS) and ethanol production**. (A) Monosaccharide composition of PS hydrolysate. (B) Ethanol production using 20 g/l of glucose, mannose and xylose. (C) Time course of monosaccharide and ethanol production from a mixed carbon source of 8.5 g/l glucose and 1.5 g/l mannose. Symbols in (C): closed circles = glucose; open circles = mannose; open triangles = dry cell weight (DCW); closed triangles = ethanol. Error bars in (C) denote 5% error of three repeated experiments.

The monosaccharides contained in the enzymatic hydrolysate of PS are glucose, xylose, mannose and arabinose (Figure 2A). Other monosaccharides were present in negligible concentrations (< 1%). When 20 g/l of glucose, mannose and xylose were used as carbon sources, ethanol production from glucose and mannose were 8.60 g/l and 8.46 g/l, respectively (Figure 2B). The *Y*_e/glc _of glucose and mannose were 93 and 92%, respectively. The *S. cerevisiae *TJ14 strain did not consume xylose for ethanol production, and the ethanol concentration generated from xylose was almost 0 g/l (Figure 2B). Although xylose is consumable, the ethanol yield is considerably lower than that obtained from glucose [4]. When a 10 g/l of glucose and mannose mixture at ratio of 85:15 was used as the carbon source, the ethanol concentration was 5.1 g/l and the *Y*_e/glc _was 87.8% (Figure 2C), which corresponds to a 88% theoretical ethanol yield [22]. When the initial glucose concentration was less than 150 g/l in the culture of *S. cerevisiae *TJ14, the *Y*_e/glc _was 88% to 94% (g/g) (data not shown).

### Enzymatic hydrolysis of untreated PS using cellulase from PS origin

**Figure 3:**
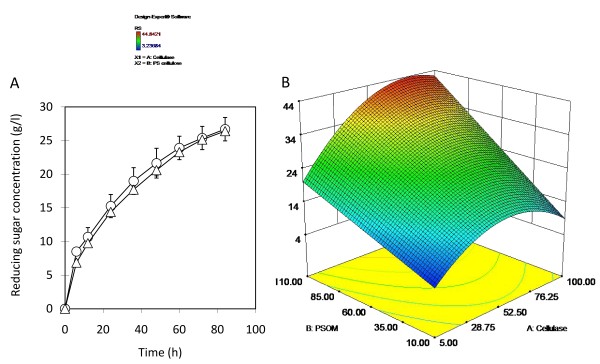
**Comparison of hydrolysis between cellulase from paper sludge (PS) and commercial Acremozyme cellulase**. **(A) **Reducing sugars (RS) concentration in PS hydrolysate when using cellulase of PS origin (open circles) and commercial cellulase (open triangles). **(B) **Surface response of the released RS concentration in an enzymatic hydrolysis of PS using cellulase of PS origin. Error bars in (A) denote 5% error of three repeated experiments.

To compare the hydrolytic performance between Acremonium cellulase (commercial origin) and cellulase produced from PS (PS origin), PS was hydrolysed using the same enzyme activity. Cellulase from PS origin was produced using PS in fermenter and obtained 900 ml of supernatant containing cellulase, of which activity was 9 FPU/ml (data not shown). The difference in RS concentration between using cellulase of PS origin and using commercial cellulase was negligible (Figure 3A) and its *Y*_s _was 54% (g RS/g PSOM). This result shows that the cellulase from PS origin performs similarly to commercial cellulase and can be used for the enzymatic hydrolysis of PS. The optimum conditions found for obtaining the highest RS concentration were 110 g PSOM/l with 80.28 FPU/g PSOM (Figure 3B). The RS prediction was 43.79 g/l with a 95% prediction interval (PI), 33.49 g/l in low and 54.09 g/l in high. According to analysis of variance (ANOVA) analysis, the model is significant. In order to confirm this prediction hydrolysis of PS was conducted on a flask scale using 110 g PSOM with 66 FPU/g PSOM. The concentrations of RS and glucose increased to 45.21 g/l and 29.2 g/l, respectively, and the *Y*_s _was 41.1% (g RS/g PSOM). These values are in the range of the optimized prediction.

### Comparison of ethanol production between SHF and SSF

**Figure 4:**
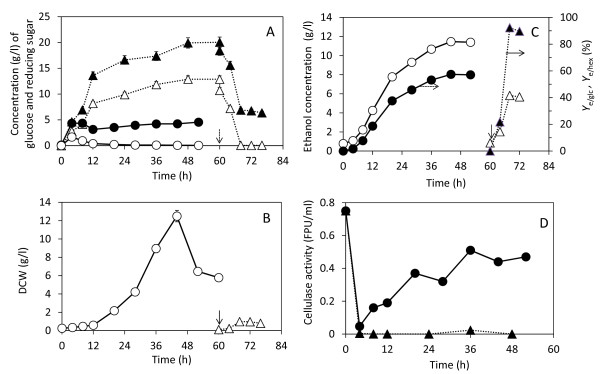
**Comparison of glucose and reducing sugars (RS) (A), dry cell weight (DCW) (B), ethanol concentration and ethanol yields, *Y*_e/hex _or *Y*_e/glc_, (C) and remaining cellulase activity (D) in separate hydrolysis and fermentation (SHF) and simultaneous saccharification and fermentation (SSF)**. A total of 50 g/l paper sludge organic material (PSOM) and 15 FPU/g PSOM were used for ethanol production. In the case of SHF the saccharification was stopped at 60 h (arrow) and initiated ethanol production by inoculation of 10% preculture of *S. cerevisiae *TJ14. Symbols in (A): closed triangles = RS of SHF; open triangles = glucose of SHF; closed circles = RS of SSF; open circles = glucose of SSF. Symbols in (B): open circles = DCW of SSF; open triangles = DCW of SHF. Symbols in (C): open circles = ethanol of SSF; open triangles = ethanol of SHF; closed circles = *Y*_e/hex _of SSF; closed triangles = *Y*_e/glc _of SHF. Symbols in (D): closed circle = cellulase activity in SSF; closed triangles = cellulase activity in SHF. Solid and dotted lines denote SSF and SHF, respectively. Error bars in (A) and (B) denote 5% error of three repeated experiments.

SHF was carried out using conditions of 50 g/l of PSOM with 15 FPU/g PSOM cellulase. After 48 h of saccharification, RS and glucose concentrations were 20 and 12.9 g/l, respectively (Figure 4A). Yeast inoculation (10%) then initiated fermentation at a culture time of 60 h; at this time glucose was diluted as a result of the inoculum, which was 10.7 g/l. The DCW of the yeast was below 1 g/l (Figure 4B) and the ethanol concentration produced was 5.02 g/l (Figure 4C). The *Y*_e/glc_, *Y*_e/hex_, and *Y*_e/psom _were 92.1% (g ethanol/g consumed glucose), 26.9% (g ethanol/g initial hexose) and 10.0% (g ethanol/g PSOM), respectively.

In SSF, under conditions of 50 g/l PSOM with 15 FPU/g PSOM cellulase and 10% inoculum, the glucose and RS concentrations increased up to 4 h (Figure 4A). During the subsequent time period, the DCW increased to 0.6 g/l at 12 h and reached 12 g/l at 44 h (Figure 4B). The glucose concentration was found to be almost 0 g/l (Figure 4A), indicating that saccharification was the limiting step in ethanol production. The ethanol concentration reached 11.4 g/l at 44 h (Figure 4C). The maximum *Y*_e/hex _and *Y*_e/psom _were 57.4% and 21.4%, respectively.

The cellulase activity was investigated at SHF and SSF. In SHF, the glucose or RS concentration was higher than 8 g/l and 14 g/l at 12 h. High RS or glucose concentration might cause deactivation of cellulase [23,24] (Figure 4D) because the hydrolysate rate decreased after that. However, during SSF, the enzyme activity remained at around 60% of initial activity (Figure 4D). However, the activity dropped below 10% of the initial activity before 4 h (Figure 4D). Initially, the glucose concentration was below 5 g/l and therefore did not deactivate cellulase, but insoluble materials contained in PS, for example clay and cellulose, adsorbed the cellulase. Since cellulase activity was assayed only in the supernatant, the cellulase adsorbed on the surface of cellulose and clay was excluded from the cellulase assay. Therefore, in the first 4 h, the measured cellulase activity was very low. However, at subsequent timepoints, with the progress of the hydrolysis of PSOM the cellulase detached from the surface of PSOM and insoluble materials and released to supernatant. As a result, the cellulase activity recovered.

These results show that SSF was preferable for ethanol production from PS. A method of semi-SSF that consisted of prehydrolysis and SSF was found to be unsuitable for this process, because of the long saccharification time and remaining high glucose concentration during reaction [6].

### Improved ethanol production in SSF

**Figure 5:**
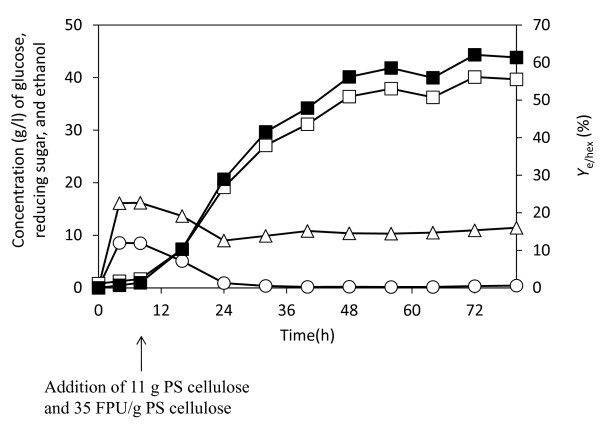
**Confirmation of optimized ethanol production in simultaneous saccharification and fermentation (SSF)**. Symbols: open triangles = reducing sugars (RS); open circles = glucose; open squares = ethanol; closed squares = *Y*_e/hex_. Error bars denote 5% error of three repeated experiments.

In order to maximize ethanol concentration from PS, the PSOM amount (50-110 g/l) and cellulase activity (15-35 FPU/g PSOM) were optimized. Surface response (Expert design v. 7.1.6) showed ethanol production trends (Additional file 1) following the equation below:

$$C_{e}=-1.43119+0.23436\times C_{psom}+0.11351\times E_{0}$$

where *C*_psom _and *E*_0 _denote PSOM concentration and initial cellulase activity, respectively. ANOVA analysis was significant for the model and both parameters gave (Prob > F) less than 0.0001 for the model and the PSOM parameter, and (Prob > F) = 0.0268 for the cellulase parameter. From this simulation, experimental conditions of 170 g/l PSOM and 35 FPU/g PSOM were predicted to give the maximum ethanol concentration of 42.38 g/l with 90% Prediction Interval (39.01 g/l for low and 45.75 g/l for high). This was confirmed by experimental data. Since PSOM concentrations of more than 110 g/l cause problems with mixing, SSF was carried out using initial conditions with 126.9 g PSOM/l, and 8.5 PSOM g was then added at 8 h. Finally, the PSOM concentration was 170 g PSOM/l with cellulase of 35 FPU/g PSOM. The ethanol concentration reached 40.10 g/l under these conditions (Figure 5) and the *Y*_e/hex _was 62.5%, which is within the range predicted by the model.

**Figure 6:**
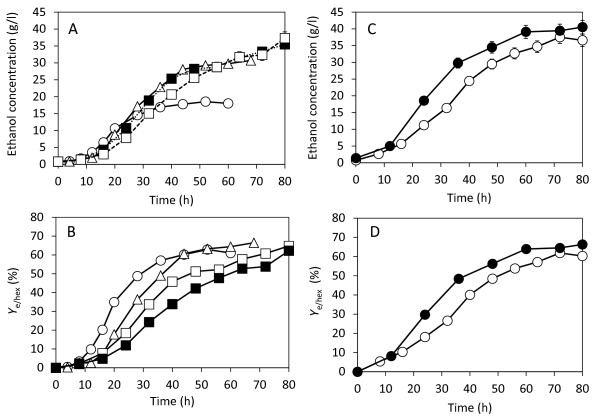
**Simultaneous saccharification and fermentation (SSF) with increased paper sludge organic material (PSOM) concentration (A) and the theoretical ethanol yield *Y*_e/hex _(B), and SSF with increased inoculum size (C) and the theoretical ethanol yield *Y*_e/hex _(D)**. (A, B) Initial conditions were 80 g PSOM/l and 15 FPU/g PSOM (open circles). PSOM (11 g, or 44.9 g PS) with PS cellulase 15 FPU/g PSOM was then added at 8 h (open triangles), at 8 h and 16 h (open squares), and at 8 h, 16 h and 20 h (closed squares). (C, D) Ethanol production increased inoculum size when 100 g PSOM/l and 15 FPU/g PSOM was used and with the addition of 11 g PSOM with 15 FPU/g PSOM at culture times of 8 and 16 h. Symbols: open circles = 10% v/v inoculum; closed circles = 20% v/v inoculum. Error bars in (A) denote 5% error of three repeated experiments.

In order to minimize the amount of cellulase required, addition of PS and increasing the inoculum were tested. When 80 g/l PSOM and 15 FPU/g PSOM cellulase were used, 18.5 g/l of ethanol was obtained. To avoid depletion of PSOM, it was added in portions of 11.0 g PSOM (44.9 g PS) up to three times, resulting in final PSOM concentrations of 127, 151 and 165 g PSOM/l with 15 FPU/g PSOM cellulase, respectively. Ethanol concentrations achieved under these conditions were 30.7, 35.7 and 37.2 g/l, respectively (Figure 6A), of which theoretical ethanol yields (*Y*_e/hex_) were 63.0, 61.8, and 59.0%, respectively (Figure 6B). No significant improvement was achieved when the addition was carried out two or three times. This suggests that the yeast concentration limited ethanol fermentation.

To solve this problem, the amount of inoculum used was increased to 20%, with an initial PSOM concentration of 100 g/l, and two additions of 11.0 g PSOM (total PSOM concentration: 161 g/l). The ethanol concentration produced under these conditions increased from 35.7 to 40.5 g/l (Figure 6C) and *Y*_e/hex _improved to 66.3%. This process did not improve the ethanol production significantly. This was also confirmed in the 2 l Erlenmeyer flask with a working volume of 1.2 l, and the ethanol concentration reached 38.8 g/l with *Y*_e/hex _of 63.4% at a culture time of 72 h (data not shown).

## Discussion

**Figure 7:**
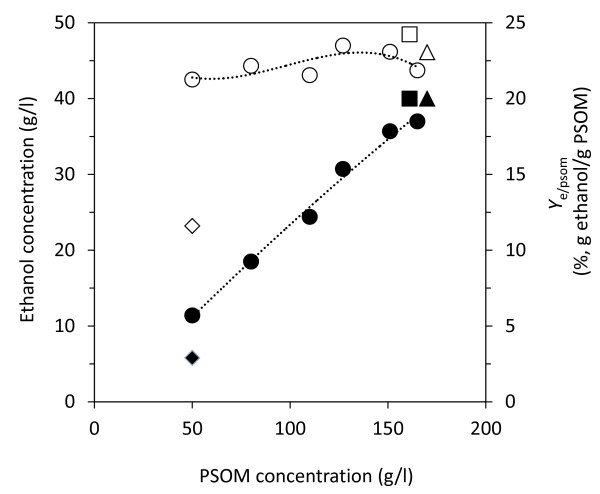
**Ethanol yield (*Y*_e/psom_) and concentration in simultaneous saccharification and fermentation (SSF) for various paper sludge organic material (PSOM) concentrations**. Data are cited from Figures 3-5. Symbols: closed circle = ethanol concentration; open circles = *Y*_e/psom_. Open and closed triangles denote *Y*_e/psom _and ethanol concentration, respectively, when increased PSOM with 35 FPU/g PSOM was used. Open and closed squares denote *Y*_e/psom _and ethanol concentration, respectively, when a 20% inoculum was used. Open and closed rhombuses denote *Y*_e/psom _and ethanol concentration of separate hydrolysis and fermentation (SHF), respectively.

This study establishes a method for practical ethanol production from PS without any pretreatment. A process that produces a high ethanol concentration with a high ethanol yield from PS was targeted. When 50 g/l of PSOM was used, the *Y*_e/psom _of SHF and SSF were 9.9 and 21.3% (g ethanol/g PSOM), respectively, but the ethanol concentration with SSF was 11.4 g/l. However, when the PSOM concentration was increased the ethanol concentration reached nearly 40 g/l. The ethanol concentration improved with increasing PSOM concentration, and the *Y*_e/psom _remained 24% (g ethanol/g PSOM) up to concentrations of 150 g PSOM/l (Figure 7). At PSOM concentrations higher than 160 g/l in SSF, however, the process was hindered by mass transfer limitation. PSOM constitutes only 24.5% of PS, meaning that 160 g PSOM is equivalent to 653 g PS/l. It was found to be impossible to mix 653 g/l of PS homogeneously, leading to decreased enzymatic hydrolysis performance. When 140 g/l of PSOM was used initial ethanol productivity (up to 8 h) decreased around 40% compared to that using 80 g/l of PSOM (Additional file 2). This is evident from the decreased *Y*_e/psom _observed at 165 g/l of PSOM. Higher ethanol concentration, more than 170 g/l of PS should be handled.

In order to increase ethanol concentration, the PSOM concentration must be increased. To increase ethanol concentration to 40 g/l, two strategies were devised: increasing cellulase activity to solve glucose limitation, and increasing the fermentation inoculum to improve ethanol production. The cellulase activity was increased to 35 FPU/g PSOM to increase saccharification yield by around 5%. The ethanol concentration increased from 37 g/l to 40 g/l and the *Y*_e/psom _also increased from 21 to 24% (g ethanol/g PSOM). When 20% of the inoculum was used, the ethanol concentration, *Y*_e/psom_, and *Y*_e/hex _increased to 40.5 g/l, 24.2%, and 66.3%, respectively. This result was similar to that of bioconversion of Kraft paper mill sludge to ethanol using SSF [24]. Ideal ethanol production from cellulose was observed for SF, since SF consists entirely of cellulose. *Y*_e/cellulose _using 50 g SF/l was 20.3% (data not shown), which is the same yield as that obtained using 50 g PSOM cellulose/l in SSF. Therefore, SSF of PS can be considered to be nearly the same as the ideal process using SF as carbon source.

**Figure 8:**
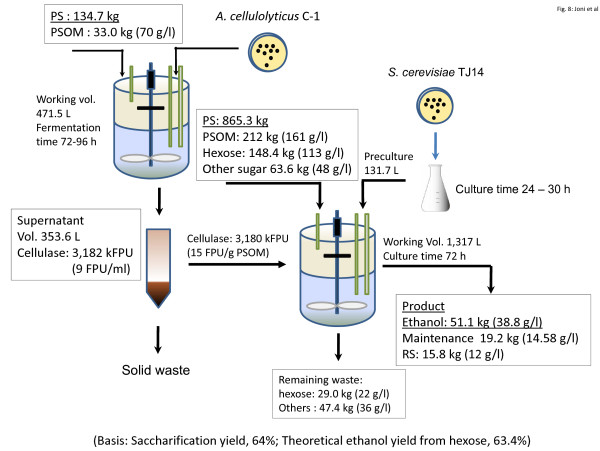
**Mass balance for cellulase production and ethanol production using 1000 kg of paper sludge (PS)**. A total of 135 kg and 865 kg of PS were used for cellulase and ethanol production, respectively. Theoretical ethanol yields *Y*_e/hex _of 63.4% and the yield from PS organic material (PSOM) *Y*_e/psom _of 24% were based for estimation of ethanol production, respectively. Since it is impossible to measure glucose concentration during simultaneous saccharification and fermentation (SSF), *Y*_s _was estimated 64% based on experimental data (38.8 g/l of ethanol production) in a 1.2 l reactor, as shown in Table 3.

Based on the 2 l scale results (*Y*_e/hex _of 63.4%), a simple mass balance for ethanol production from PS was estimated (Figure 8). When 1 ton of PS is used in ethanol production, around 134.7 kg of PS is used for cellulase production. This cellulase is then used for the enzymatic hydrolysis of another 865.3 kg of PS and bioconverts PSOM to ethanol. The ethanol production is predicted to be about 51.1 kg based on *Y*_e/psom _of 23.5% (g ethanol/g PSOM). In Japan, around 5 million tons of PS is discarded annually, and if this amount were to be used for bioethanol production 255,000 tons of ethanol could be produced. A recent trend in automotive fuels involves the blending of ethanol (5% to 10%) with gasoline [25,26], as this allows the present fuel distribution infrastructure to be used largely unchanged. A total of 255,000 tons of ethanol could be blended at the 5% level with 5,100,000 tons of gasoline. The results of ethanol production from PS using PS cellulase produced by *A. cellulolyticus *described here demonstrates the potential of this process for future bioethanol production. Further studies are planned to allow ethanol production from PS to be scaled up.

**Table 3:** *Y*_s _estimated 64% based on experimental data; 192 g PSOM used

Factor	Estimated sugar amount (g)
Theoretical glucose needed	38.8 g/l/0.51 × 1.2 l = 91.3
RS at the end fermentation	11 g/l × 1.2 l = 14.4
Glucose for yeast maintenance	*m*^a ^(g/g cell/h) × 7.1 g/l × 72 h = 17.5
Total RS in hydrolysate	123
Estimated *Y*_s _(%)	123/192 × 100 = 64.0

^a^Where *m *= 0.034 g glucose/(g cell/h) [19].

PSOM = paper sludge organic material; RS = reducing sugars.

## Conclusions

The ethanol yield (*Y*_e/psom_) obtained when 50 to 150 g PSOM/l was used was 21% to 23% (g ethanol/g PSOM) in the SSF, which is two times higher than that obtained using SHF. Cellulase activity remained at around 60% throughout SSF. Within the PSOM concentrations less than 160 g PSOM/l, the ethanol yield remained at 23% with the ethanol concentration of 40 g/l. Ethanol production of 40 g/l was achieved using 161 g/l of PSOM with 15 FPU/g PSOM and 20% inoculum, after 80 h using the optimized SSF process. This was confirmed in the 2 l scale experiment and indicates that there is great potential to use PS as a raw material for ethanol production.

## Competing interests

The authors declare that they have no competing interests.

## Authors' contributions

JP was responsible for the experimental design, and the hydrolysis and fermentation experiments. KN participated in measurement of cellulase activity and PS saccharification. HS and CB provided *Saccharomyces cerevisiae *TJ14. EYP directly supervised the project, participated in its experimental design, data interpretation, and was responsible for writing the manuscript. All authors read and approved the manuscript.

## Supplementary Material

Additional file 1 **Optimization of cellulase and paper sludge organic material (PSOM) concentration for improving ethanol concentration**. PSOM concentration and cellulase activity were optimized under the following conditions: initial PSOM concentrations were 50, 80 and 110 g/l and cellulase activities were 15, 25 and 35 FPU/g PSOM. After medium sterilization, the cellulase and 10% inoculum were added to 500 ml Erlenmeyer flasks with final working volumes of 100 ml. Data were analyzed by Design Expert (v. 7.1.6, Stat-Ease, Minneapolis, MN, USA). Click here for file

Additional file 2 **paper sludge (PS) appearance during simultaneous saccharification and fermentation (SSF)**. **(A)**, **(B)**, and **(C) **denote PS appearance during SSF at 0, 4, and 8 h, respectively. (1) and (2) indicate PS organic material (PSOM) concentrations of 80 and 140 g/l, respectively. When 140 g/l of PSOM was used it is impossible to mix culture broth. **(D) **Effect of initial PSOM concentration on ethanol production rate until 8 h. Click here for file
