# Supplementary figures and images for: Bioconversion of paper sludge to biofuel by simultaneous saccharification and fermentation using a cellulase of paper sludge origin and thermotolerant Saccharomyces cerevisiae TJ14
Source: Biotechnol Biofuels. 2011 Sep 29;4:35. doi: 10.1186/1754-6834-4-35 (PMC3203334; doi:10.1186/1754-6834-4-35)

## Slide 1
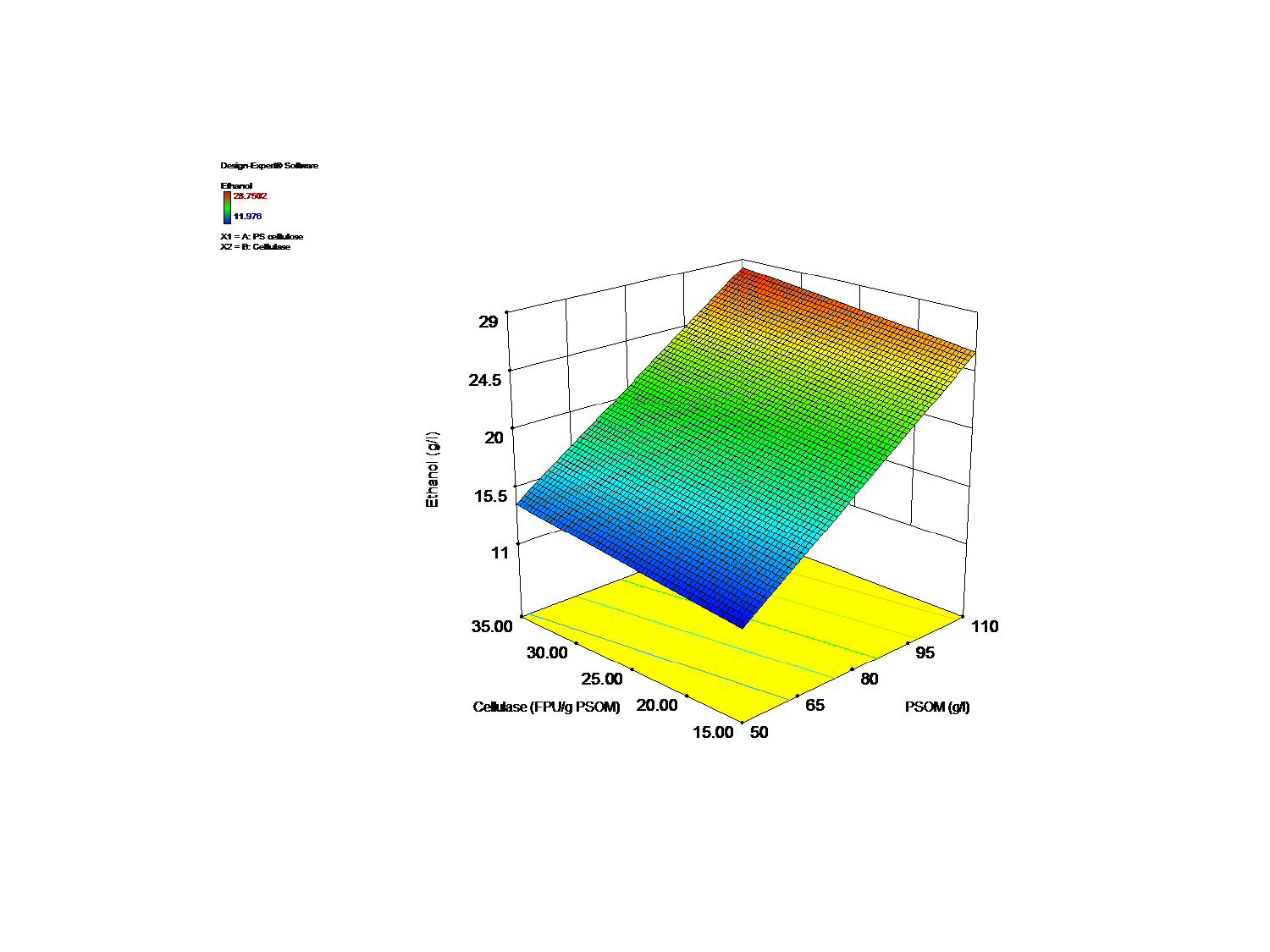

Supplement: Additional file 1 — Optimization of cellulase and paper sludge organic material (PSOM) concentration for improving ethanol concentration. PSOM concentration and cellulase activity were optimized under the following conditions: initial PSOM concentrations were 50, 80 and 110 g/l and cellulase activities were 15, 25 and 35 FPU/g PSOM. After medium sterilization, the cellulase and 10% inoculum were added to 500 ml Erlenmeyer flasks with final working volumes of 100 ml. Data were analyzed by Design Expert (v. 7.1.6, Stat-Ease, Minneapolis, MN, USA). [file 1754-6834-4-35-S1.PPT]

## Slide 1
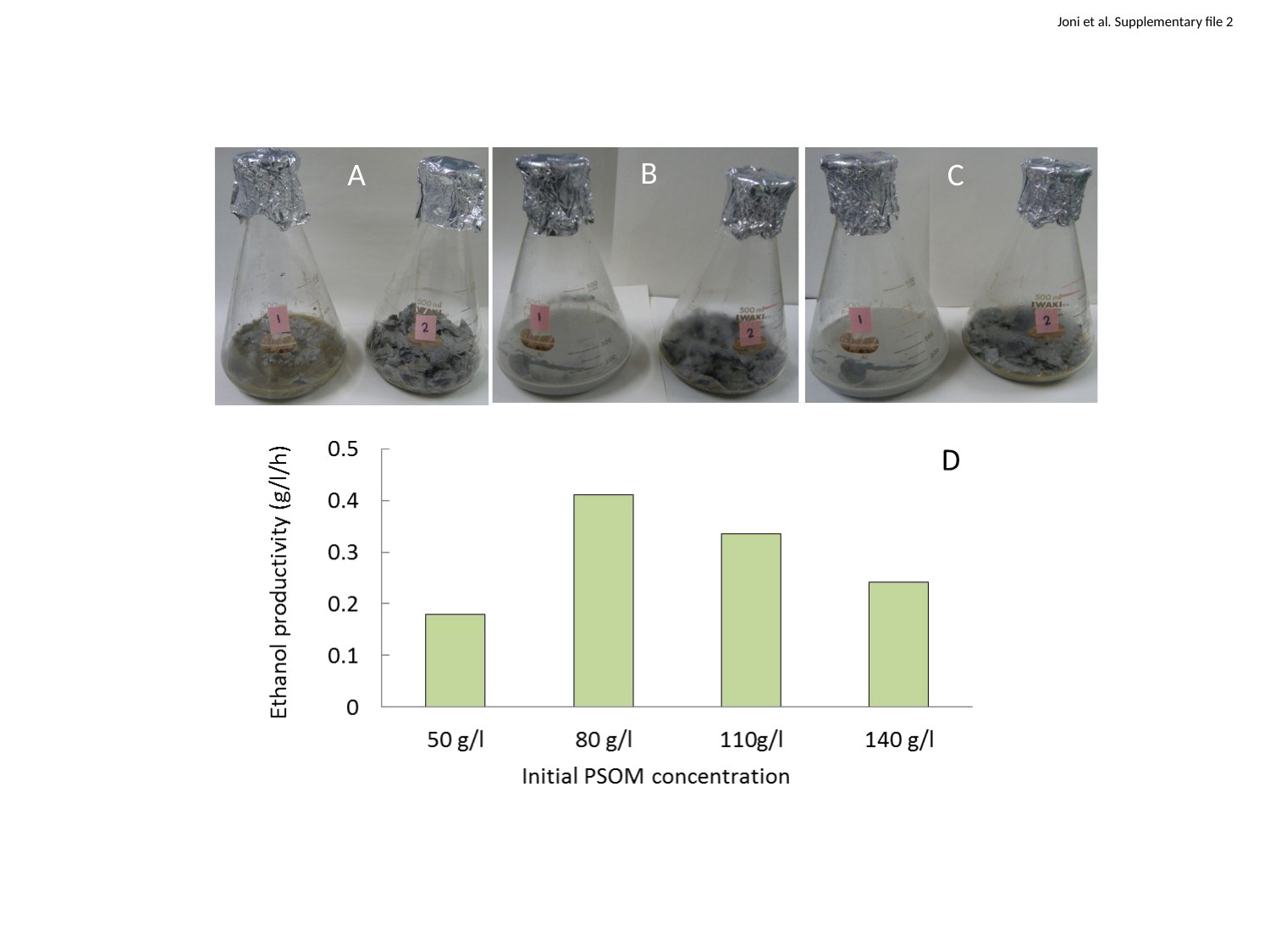

Joni et al. Supplementary file 2
B
A
C

Supplement: Additional file 2 — paper sludge (PS) appearance during simultaneous saccharification and fermentation (SSF). (A), (B), and (C) denote PS appearance during SSF at 0, 4, and 8 h, respectively. (1) and (2) indicate PS organic material (PSOM) concentrations of 80 and 140 g/l, respectively. When 140 g/l of PSOM was used it is impossible to mix culture broth. (D) Effect of initial PSOM concentration on ethanol production rate until 8 h. [file 1754-6834-4-35-S2.PPT]
